# Mindfulness-Oriented Professional Resilience (MOPR) Training to Reduce Compassion Fatigue in Healthcare Workers: A Pilot Study

**DOI:** 10.3390/healthcare13020092

**Published:** 2025-01-07

**Authors:** Fabio D’Antoni, Alessio Matiz, Cristiano Crescentini

**Affiliations:** 1 Maternal Infant Services Unit of Udine, Azienda Sanitaria Universitaria Friuli Centrale (ASUFC), 33100 Udine, Italy; 2 Department of Psychology, Sapienza University of Rome, 00185 Rome, Italy; alessio.matiz@uniud.it; 3 Department of Languages and Literatures, Communication, Education and Society, University of Udine, 33100 Udine, Italy; cristiano.crescentini@uniud.it

**Keywords:** healthcare professionals, compassion fatigue, compassion satisfaction, mindfulness, traumatic stress, resilience, arousal modulation, polyvagal theory, acceptance and commitment therapy (ACT), self-compassion

## Abstract

**Background/Objectives**: Compassion Fatigue (CF) is a critical issue among healthcare professionals, exacerbated by exposure to trauma and chronic workplace stress. This pilot study evaluates the effectiveness of a Mindfulness-Oriented Professional Resilience (MOPR) program, a structured intervention designed to mitigate CF and enhance resilience in healthcare professionals. The program integrates mindfulness practices, arousal modulation techniques, and resilience-building strategies over six weekly sessions. **Methods**: A sample of 73 healthcare workers (mean age 48.6, SD = 9.42) participated in the study, and pre–post data were analyzed using repeated measures ANOVA. **Results**: Results indicated significant improvements in professional quality of life, with increased Compassion Satisfaction (*p* < 0.001) and reductions in Burnout (*p* = 0.003) and Secondary Traumatic Stress (*p* < 0.001). Mindfulness skills improved significantly across four dimensions—Observing, Describing, Acting with Awareness, and Non-reactivity—with *p*-values ranging from <0.01 to <0.001. Arousal modulation showed increased Optimal Arousal Zone scores (*p* < 0.001) and reduced maladaptive stress responses, including Fight/Flight, Freeze, and Feigned Death (*p* < 0.05). Psychological well-being improvements were observed, particularly in Self-Acceptance (*p* = 0.014) and Positive Relations (*p* = 0.041). **Conclusions**: These findings suggest that the MOPR program is a promising intervention for healthcare professionals, supporting resilience and reducing the psychological burden of caregiving. Future controlled studies should explore its long-term efficacy in diverse clinical settings and in larger samples.

## 1. Introduction

The term “Compassion Fatigue” was first coined by Carla Joinson [1], and it has been widely discussed in the context of healthcare and caregiving professions [2]. Much of the literature on the subject, however, relies on the definition provided by Charles Figley (1995), who described Compassion Fatigue as the “cost of caring” for individuals in emotional or physical pain [3]. According to Figley (1999), the term referred to a state of emotional and physical exhaustion that arose from prolonged exposure to the suffering of others, leading to a diminished capacity to empathize and engage effectively with patients [4]. Often conceptualized as a Secondary Traumatic Stress reaction, it was distinguished from Burnout due to its direct link with trauma exposure through empathetic engagement [5].

To further investigate the psychological impact of caregiving on healthcare professionals, Stamm’s Professional Quality of Life Model (ProQOL) provided a comprehensive framework by identifying two key components: Compassion Satisfaction (CS) and Compassion Fatigue (CF) [6]. CS referred to the positive emotional outcomes that caregivers derived from their work, offering a buffer against the emotional strain associated with continuous exposure to suffering and stress. CF, on the other hand, encompassed both Secondary Traumatic Stress (STS) and Burnout (BO). STS reflected the distress resulting from empathetic engagement with individuals experiencing trauma, while BO was characterized by emotional exhaustion stemming from chronic workplace stress [7]. Importantly, BO was distinguished from CF by its etiology: while CF directly related to trauma exposure through caregiving, BO arose from broader, long-term responses to work-related stressors that may not have involved direct contact with trauma [6,7].

CF remains a prevalent issue across various healthcare professions, particularly those that involve high levels of emotional engagement and exposure to patient suffering. Nurses, who often serve as frontline caregivers, are among the most affected due to their frequent and direct interactions with patients in distress [8,9,10]. Studies showed that nurses working in critical care, oncology, and emergency departments were especially vulnerable to CF, given the intensity and emotional demands of these settings [11,12,13,14,15]. Similarly, physicians, particularly those in palliative care, trauma, and emergency medicine, also reported high levels of CF, as they were regularly confronted with patient suffering, death, and complex emotional needs [16,17,18]. Mental health professionals, such as psychologists and social workers, are not immune to CF either, as their work often involves managing the psychological trauma of others, leading to STS [19,20,21,22,23]. The COVID-19 pandemic has further exacerbated CF across healthcare settings, placing unprecedented psychological strain on professionals due to increased workloads, high patient mortality, and the prolonged emotional intensity of the crisis [8,20,23,24,25,26]. However, even before the COVID-19 pandemic, several studies had already highlighted the critical state of workplace well-being among various healthcare professionals, including physicians and anesthesiologists [27,28,29].

This widespread occurrence of CF across various healthcare fields highlights the urgent need for preventative strategies and resilience-building programs to support healthcare workers in maintaining their well-being while continuing to deliver compassionate care [30]. In response to this need, several programs have been developed to mitigate CF, including mindfulness-based interventions, resilience training, and psychoeducational and reflective practices [31,32,33,34,35,36]. These programs aim to enhance coping mechanisms, promote self-care, and increase Compassion Satisfaction, ultimately helping healthcare professionals to manage the emotional demands of their work more effectively [37,38,39]. Recent evidence suggests that psychological interventions can effectively reduce Compassion Fatigue among helping professionals, providing critical support in mitigating its impact on mental health and job performance [40].

The present study directly addresses the gaps identified in existing CF interventions, particularly the need for more tailored, evidence-based programs aimed at healthcare professionals. Although several interventions, including mindfulness-based and resilience-building programs, have been implemented to reduce CF [41,42,43], many lack specific attention to the component of Secondary Traumatic Stress (STS) [44]. This form of stress is particularly pervasive in healthcare settings where professionals frequently encounter patient trauma and suffering, especially during the COVID-19 pandemic [44], which has significantly exacerbated these challenges.

Building on prior research and interventions, the current study aims to develop and evaluate a Mindfulness-Oriented Professional Resilience (MOPR) training program designed to foster resilience among healthcare workers. The primary focus of this program is not only to reduce overall CF but to address the psychological burden of STS more specifically. The program aims to enhance healthcare workers’ mindfulness skills, improving their emotional regulation and awareness to better manage distressing situations. It also fosters emotional and arousal regulation, helping them handle the intense demands of their work while reducing emotional exhaustion. Additionally, it provides sustainable, practical tools that can be easily integrated into daily routines, ensuring long-term benefits in managing psychological challenges. By focusing on both CF and STS, this study aimed to contribute to the growing body of literature on healthcare worker resilience, offering a targeted, evidence-based approach that can be scaled and adapted across various healthcare settings. Moreover, the comprehensive design of the MOPR training combines bottom-up components, such as the cultivation of mindfulness-based mental states through meditation and arousal self-regulation exercises, with top-down approaches, including guided reflection and psychoeducation. The hypothesis is that this integrative framework enables participants to engage with the training’s elements in a manner that best aligns with their individual needs, functioning, and personal inclinations, thereby maximizing the potential for meaningful and sustained improvements across interconnected domains. These domains, which include professional quality of life, mindfulness skills, arousal modulation, and psychological well-being, are measured pre- and post-intervention and are hypothesized to be directly influenced by the activities and practices incorporated in the training. In particular, we expect that these activities will converge in improving arousal regulation, thereby reducing Compassion Fatigue and potentially enhancing Compassion Satisfaction.

## 2. Materials and Methods

### 2.1. The Program

The MOPR program was developed by integrating over 15 years of the first author’s clinical experience in the treatment of psychological trauma with insights from the existing literature on mindfulness-based approaches. Key influences included established mindfulness programs such as Mindfulness-Based Stress Reduction (MBSR) [45] and Mindfulness-Oriented Meditation (MOM) [46], as well as principles from the Accelerated Recovery Program (ARP) [43,47]. Unlike other interventions, MOPR combines mindfulness-based strategies with physiological self-regulation techniques to directly target autonomic nervous system responses, thereby offering a holistic approach to reducing Compassion Fatigue.

Table 1 provides an overview of the program’s core themes, detailing the theoretical frameworks and intervention models that underpin each focus area.

Mindfulness frameworks offer oriented approaches to enhance self-awareness and emotional regulation [45,46]. These practices are foundational in the MOPR program to help participants develop non-reactive awareness, which is crucial for managing CF. Mindfulness practices improve attention to present-moment experiences, which can counteract habitual stress responses.

Acceptance and Commitment Therapy (ACT) principles inform the program’s guided reflection component, which encourages participants to explore their values, goals, and workplace challenges [50,51]. By focusing on acceptance, committed action, and value-driven behavior, ACT facilitates a deeper understanding of personal and professional values, aiding participants in developing resilience and flexibility in response to workplace stressors.

Self-compassion is integrated into the program to encourage a kinder, more understanding attitude towards oneself, particularly when facing professional stressors and challenges [52]. Through self-compassion practices, participants learn to acknowledge their own suffering with empathy, which is essential for reducing self-criticism and building resilience.

The ProQOL model serves as a foundation for the MOPR program, distinguishing between Compassion Satisfaction (CS) and Compassion Fatigue (CF), with the latter encompassing Secondary Traumatic Stress (STS) and Burnout (BO) [3,7]. This model directs the program’s focus on enhancing CS while reducing CF and STS, which are prevalent among healthcare workers. ProQOL guides the program’s emphasis on resilience-building to support emotional well-being in high-stress caregiving environments.

Polyvagal Theory informs the physiological component of the program, explaining how autonomic responses, including Fight/Flight/Freeze reactions, can be modulated through vagal activation [54]. The MOPR program incorporates principles from Polyvagal Theory by teaching participants practices to stimulate the vagus nerve, promoting self-regulation and resilience in response to trauma exposure.

The arousal modulation model from Sensorimotor Psychotherapy [53] is incorporated to guide participants in recognizing and managing their physiological arousal levels, supporting them in maintaining an Optimal Arousal Zone, which is essential for preventing emotional exhaustion.

Heart Rate Variability (HRV) biofeedback principles are incorporated to improve participants’ autonomic flexibility and vagal tone, enhancing their ability to self-regulate under stress [55,56].

Finally, participants create a personalized resilience plan, similar to the approach used in the Accelerated Recovery Program (ARP) [43,47], which integrates all themes of the MOPR program. This plan enables participants to consolidate and apply the skills and insights gained from each session, supporting sustainable self-care practices and enhancing long-term resilience.

The MOPR program was structured as an educational intervention that extends beyond knowledge acquisition, actively engaging participants in the practice of targeted techniques to manage stress, build resilience, and incorporate sustainable self-care routines into daily life. Indeed, this approach was tailored for professionals in emotionally demanding fields who regularly face high-stress and trauma-laden situations. By equipping them with knowledge and strategies to recognize and manage Compassion Fatigue, the program aims not only to enhance their understanding but also to function as a proactive measure for sustaining emotional well-being and resilience.

The MOPR program consisted of six structured group sessions, each lasting 2 h and 30 min (see Table 2). Each session was designed to include four main components: (1) psychoeducation on core topics of the program (see Table 1), (2) guided reflection on participants’ relationship with work, culminating in the development of a personalized resilience plan, (3) mindfulness meditation practices and vagal stimulation/emotion regulation exercises (including diaphragmatic breathing and targeted stretching), and (4) group sharing and discussion.

In addition, participants were encouraged to practice the techniques learned during each session at home and to document their experiences in a printed workbook. This workbook included materials from each session, provided a structured format for tracking progress throughout the program, and served as a tool for drafting their personalized resilience plans during the final session. The recommended home practice ranged from a minimum of 5 min to 40 min per day, six days a week.

Before beginning the MOPR program, participants were provided with an optical heart rate sensor for measuring Heart Rate Variability (HRV) via a smartphone application, which is essential for determining each participant’s personal resonance frequency [56]. This tool helped participants develop self-regulation skills, allowing them to observe physiological responses to stress and practice techniques to achieve optimal arousal levels. Participants were also encouraged to use the HRV sensor at their discretion, primarily to monitor diaphragmatic breathing exercises performed at their individual resonance frequency.

At the end of the sixth session, a resource map was introduced to participants, outlining available organizational support (e.g., mindfulness courses, Burnout prevention programs, psychological counseling, and personalized lifestyle consultations) to help them access resources in their workplaces that could enhance their professional quality of life. Together, these resources bridged the gap between session-based learning and real-world application, helping participants internalize and sustain skills for long-term well-being.

The trainer was a highly experienced clinical psychologist with over 15 years of specialization in psychological trauma. He held certifications as an EMDR practitioner and consultant, was extensively trained in bottom-up psychotherapeutic approaches (e.g., Sensorimotor Psychotherapy), and had substantial expertise in mindfulness-based interventions. Additionally, the trainer was a dedicated practitioner of meditation in the Tibetan Buddhist tradition, further enriching his approach to facilitating the program.

### 2.2. Study Design

The study employed a longitudinal, pre–post-intervention design to assess the effectiveness of the MOPR program on participants’ professional quality of life, mindfulness skills, arousal modulation skills, and overall psychological well-being. Participants attended six group sessions over a period of six weeks, each lasting 2.5 h. Data were collected at baseline (pre-intervention) and immediately following the final session (post-intervention) to evaluate changes across multiple psychological measures.

### 2.3. Setting and Participants

The intervention was conducted as a formal training course offered by the Azienda Sanitaria Universitaria Friuli Centrale (ASUFC), a part of the Italian public healthcare system. Enrolment was voluntary for ASUFC employees, and sessions were scheduled during regular working hours to facilitate participation. Participants received Continuing Medical Education (CME) credits upon completion of the entire program. A deliberate focus was placed on psychologists during the initial recruitment phase, as one of the program’s goals was to prepare a subset of participants for additional training to become future trainers of the intervention. While this approach ensured that the program could sustain its implementation through a “train-the-trainer” model, it may have introduced selection bias by attracting individuals with a pre-existing interest or background in psychological interventions, mindfulness practices, or related topics. The sample also included a range of other healthcare professionals, but it is important to note that the representation of these professional categories (e.g., nurses, physicians, allied health workers) was more limited in comparison to psychologists. This could influence the generalizability of the results to the broader population of healthcare workers. Sessions took place in a dedicated, quiet space within the organization, providing a supportive environment conducive to mindfulness and self-reflection, tailored to the needs of healthcare professionals. This structured approach, embedded within the work environment, encouraged the integration of program principles into participants’ daily professional and personal routines.

The participants included healthcare workers from diverse professional categories: medical doctors (8%), clinical psychologists (52%), nurses (25%), healthcare assistants (3%), rehabilitation therapists (11%), and biomedical laboratory technicians (1%). This distribution reflects a multidisciplinary composition, with most participants involved in mental health services. The program was designed to address the shared challenges faced by these professions, including Compassion Fatigue and emotional demands. Demographic and professional characteristics of the participants are detailed in Table 3.

### 2.4. Measures

We used the Professional Quality of Life Scale (ProQOL), Version 5. The ProQOL is a 30-item questionnaire designed to assess professional quality of life, focusing on both positive and negative aspects of caregiving. It measures Compassion Satisfaction (CS) and Compassion Fatigue (CF), with the latter divided into two dimensions: Secondary Traumatic Stress (STS) and Burnout (BO). Participants rate each item based on their experiences over the past 30 days using a 5-point Likert scale [6,7,57]. In the current sample, the Cronbach’s alpha values were 0.90 for CS, 0.71 for BO, and 0.82 for STS, indicating good-to-excellent internal consistency.

Further, we used the Five Facet Mindfulness Questionnaire—Short Form (FFMQ-SF). The short form of the FFMQ is a 24-item questionnaire that uses a 5-point Likert scale to assess mindfulness across five key dimensions: (1) Observing, (2) Describing, (3) Acting with Awareness, (4) Non-judging, and (5) Non-reactivity. This tool captures various facets of mindfulness, allowing a nuanced evaluation of participants’ mindfulness skills [58]. The Cronbach’s alpha values for the subscales in this sample were 0.83 for Observing, 0.84 for Describing, 0.88 for Acting with Awareness, 0.86 for Non-judging, and 0.80 for Non-reactivity, reflecting good internal consistency across all dimensions.

We also used the Arousal Modulation Model Questionnaire (AMMQ). The AMMQ is a newly developed instrument (D’Antoni et al., under review) based on the Arousal Modulation Model from Sensorimotor Psychotherapy (Ogden et al., 2023). It includes 22 items distributed across four main scales: (1) Optimal Arousal Zone (OAZ), hyperarousal reactions such as (2) “Fight/Flight” (FF) and (3) “Freeze” (Fr), and the hypoarousal response of (4) “Feigned Death” (FD). The AMMQ assesses autonomic regulation across different arousal states, providing valuable insights into the MOPR’s influence on stress reactivity and physiological resilience. The version used in this study showed good model fit indices in an independent sample of 304 adults (χ^2^ (203) = 381.738; CFI = 0.940; TLI = 0.932; RMSEA = 0.054 [90% CI: 0.045–0.062], *p* = 0.219; SRMR = 0.047) and high internal consistency (all α > 0.80). Items refer to participants’ experiences over the past month and are rated on a 5-point Likert scale. In this sample, the Cronbach’s alpha values were 0.91 for OAZ, 0.87 for FF, 0.83 for Fr, and 0.87 for FD, indicating excellent internal consistency for the subscales.

Lastly, we used the Psychological Well-Being Short Form (PWB-SF). The short form of the PWB consists of 18 items rated on a 6-point Likert scale, assessing various dimensions of psychological well-being. These dimensions include Self-Acceptance (SA), Autonomy (AU), Environmental Mastery (EM), Personal Growth (PG), Purpose in Life (PL), and Positive Relations with others (PR) [59,60]. The Cronbach’s alpha values in this sample were 0.79 for SA, 0.72 for AU, 0.77 for EM, 0.78 for PG, 0.78 for PL, and 0.61 for PR. These values indicate acceptable-to-good internal consistency, except for PR, which showed lower reliability.

### 2.5. Data Analysis

We conducted repeated measures ANOVA analyses for each scale—ProQOL, FFMQ-SF, AMMQ, and PWB-SF—to evaluate changes in Compassion Satisfaction and Fatigue, mindfulness, arousal modulation, and well-being across two time points (pre-test and post-test) within the same sample. Given the multiple comparisons across these scales and their subscales, Bonferroni correction was applied to control for Type I error, ensuring that significant results accurately reflected meaningful differences over time.

To examine the relationships between arousal regulation and professional quality of life, a series of linear regression analyses were conducted. By focusing on the difference scores (post-test minus pre-test) for each variable, we aimed to identify associations between improvements in professional quality of life and MOPR-related changes in optimal arousal modulation, providing insights into the interconnected effects of the MOPR program on these constructs.

## 3. Results

### 3.1. Demographics and Work-Related Backgrounds

The study sample comprised three distinct MOPR groups, collectively involving 73 participants with a mean age of 48.6 years (SD = 9.42), spanning from 27 to 71 years. Each group underwent an identical set of program activities (Table 1 and Table 2), which was conducted by the same facilitators to ensure consistency in delivery. The first group participated in the program during November–December 2023, while the second and third groups ran concurrently during September–October 2024. Participants were volunteers who expressed their intention to join after learning about the MOPR program through ASUFC’s periodic training plan communication. Data from all groups were analyzed as a single cohort to provide a comprehensive preliminary assessment of the MOPR program’s impact across a diverse range of healthcare professionals. The majority of participants were female and came from diverse educational and professional backgrounds, with most holding postgraduate degrees and over half working as clinical psychologists. Physical activity levels varied, with the majority identifying as either physically active or partially active. Engagement in mindfulness practices was less common, with a minority practicing regular meditation or breathing exercises. A small proportion of participants reported a mental health diagnosis or the use of psychotropic medication. Detailed participant characteristics are provided in Table 3.

### 3.2. Professional Quality of Life

A repeated measures ANOVA was conducted to examine the effect of time on Compassion Satisfaction (CS), Burnout (BO), and Secondary Traumatic Stress (STS) scores across two time points: pre-test and post-test. The results revealed a significant main effect of time on CS scores, *F*_(1, 72)_ = 16.84, *p* < 0.001, *η_p_*^2^ = 0.19. Similarly, significant reductions were observed for both BO and STS scores. The ANOVA for BO revealed a significant effect of time, *F*_(1, 72)_ = 9.21, *p* = 0.003, *η_p_*^2^ = 0.11. For STS, the results also indicated a significant reduction, *F*_(1, 72)_ = 22.57, *p* < 0.001, *η_p_*^2^ = 0.24 (Table 4).

**Figure 1:**
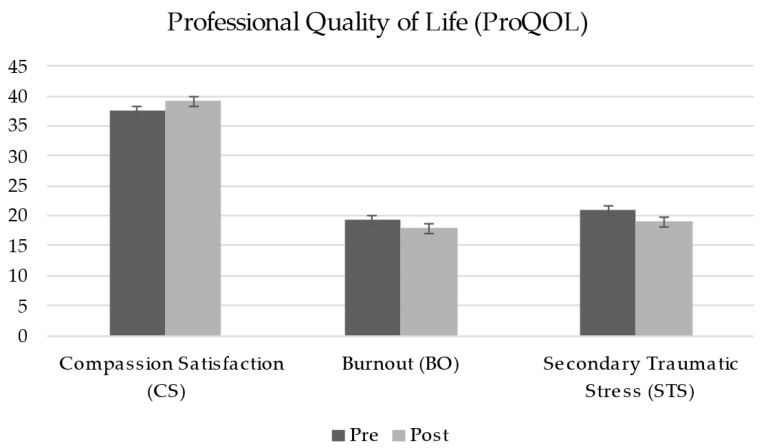
Pre-test and post-test scores for Professional Quality of Life (ProQOL).

These findings suggest that the MOPR intervention may contribute to increased Compassion Satisfaction while simultaneously reducing levels of Compassion Fatigue (Burnout and Secondary Traumatic Stress), highlighting its potential benefits for enhancing resilience among healthcare providers (see Figure 1).

### 3.3. Mindfulness

A repeated measures ANOVA was conducted to examine the effect of the MOPR program on the five facets of mindfulness, as assessed by the Five Facet Mindfulness Questionnaire—Short Form (FFMQ-SF), across two time points: pre-test and post-test. The results revealed significant main effects of time on four facets—Observing, Describing, Acting with Awareness, and Non-reacting—indicating significant increases from pre-test to post-test.

For the Observing facet, the results indicated a significant increase, *F*_(1, 72)_ = 9.13, *p* = 0.003, *η_p_*^2^ = 0.11. Similar improvements were observed for Describing, *F*_(1, 72)_ = 7.04, *p* = 0.01, *η_p_*^2^ = 0.09; Acting with Awareness, *F*_(1, 72)_ = 13.83, *p* < 0.001, *η_p_*^2^ = 0.16; and Non-reacting, *F*_(1, 72)_ = 10.77, *p* = 0.002, *η_p_*^2^ = 0.13. In contrast, the Non-judging facet did not exhibit a significant change, *F*_(1, 72)_ = 0.57, *p* = 0.454, indicating stability across time points (Table 4).

**Figure 2:**
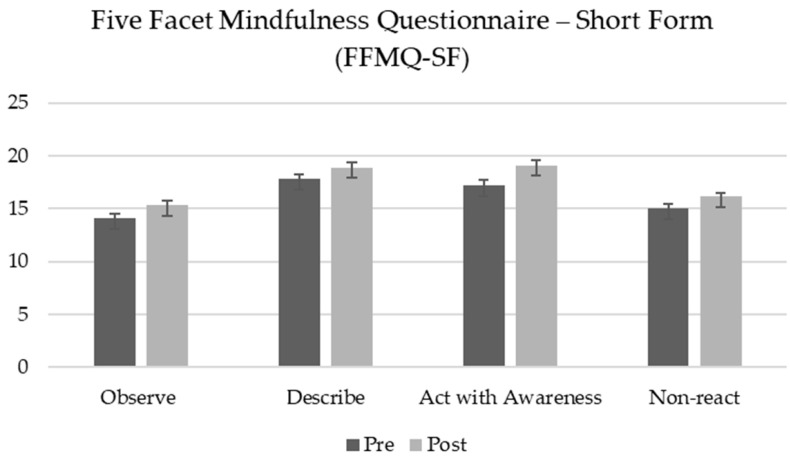
Pre-test and post-test scores for Five Facet Mindfulness Questionnaire—Short Form (FFMQ-SF). The Non-judging facet is not shown.

These results suggest that the MOPR program may effectively enhance multiple facets of mindfulness (see Figure 2).

### 3.4. Arousal Modulation

A repeated measures ANOVA was conducted to assess the effect of the MOPR program on arousal modulation and stress responses, as measured by the Arousal Modulation Model Questionnaire (AMMQ), across two time points: pre-test and post-test. Results revealed a significant main effect of time, with a marked increase in the Optimal Arousal Zone and a significant reduction in stress reactivity across the scales measuring hyperarousal (Fight/Flight and Freezing) and hypoarousal (Feigned Death) (Table 4).

Optimal Arousal Zone scores showed a significant increase from pre-test to post-test, *F*_(1, 72)_ = 22.28, *p* < 0.001, *η_p_*^2^ = 0.24. The Fight/Flight subscale, one of the hyperarousal indicators, demonstrated a significant reduction, *F*_(1, 72)_ = 11.20, *p* = 0.001, *η_p_*^2^ = 0.14. Similarly, the Freeze subscale, another hyperarousal indicator, showed a significant reduction, *F*_(1, 72)_ = 4.71, *p* = 0.033, *η_p_*^2^ = 0.06. Finally, the Feigned Death subscale, indicative of hypoarousal, showed a significant decrease from pre-test to post-test, *F*_(1, 72)_ = 5.98, *p* = 0.017, *η_p_*^2^ = 0.08.

**Figure 3:**
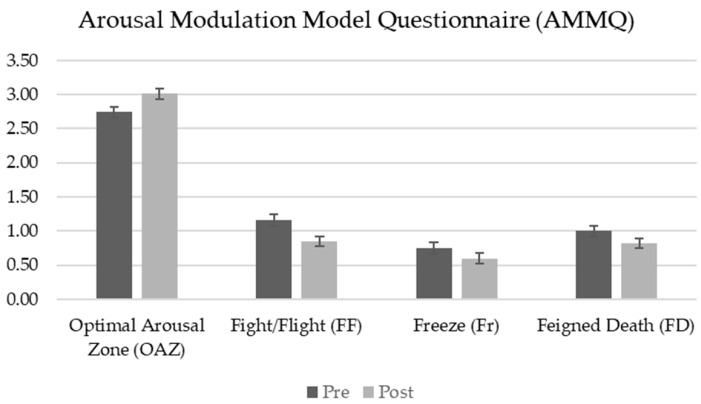
Pre-test and post-test scores for Arousal Modulation Model Questionnaire (AMMQ).

These findings suggest that the MOPR program may enhance participants’ ability to maintain an optimal arousal level and reduce maladaptive stress responses across both hyperarousal and hypoarousal domains (see Figure 3).

### 3.5. Psychological Well-Being

A repeated measures ANOVA was conducted to assess the effect of the MOPR program on psychological well-being, as measured by the Psychological Well-Being Short Form (PWB-SF), across two time points: pre-test and post-test. Results revealed an overall increase in scores across all well-being subscales; however, significant improvements were found specifically in Self-Acceptance and Positive Relations with others (Table 4).

**Figure 4:**
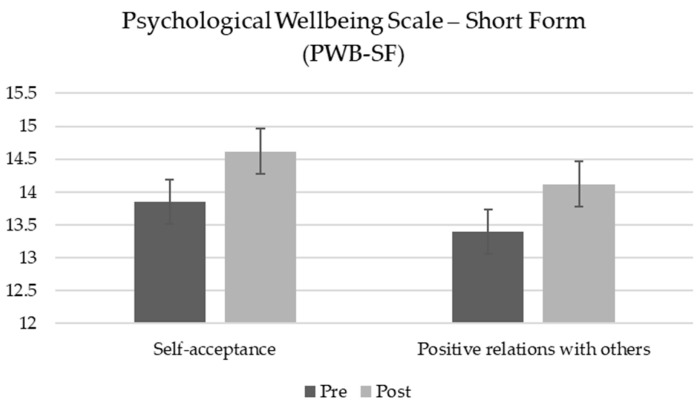
Pre-test and post-test scores for Psychological Well-Being Scale—Short Form (PWB-SF). Autonomy, Environmental Mastery, Personal Growth, and Purpose in Life data are not reported.

For the Self-Acceptance subscale, scores increased significantly from pre-test to post-test, *F*_(1, 72)_ = 6.32, *p* = 0.014, *η_p_*^2^ = 0.08. Similarly, the Positive Relations with others subscale showed a significant increase, *F*_(1, 72)_ = 4.34, *p* = 0.041, *η_p_*^2^ = 0.06. Although increases were also observed in the remaining PWB-SF subscales—Autonomy, Environmental Mastery, Personal Growth, and Purpose in Life—these changes did not reach statistical significance. These findings suggest that the MOPR program may support psychological well-being by fostering, in particular, greater Self-Acceptance and enhancing Positive Relationships (see Figure 4). All statistical results are presented in Table 4.

### 3.6. Relations Between Arousal Regulation and Professional Quality of Life

To investigate the relationships between arousal regulation and outcomes related to CF (BO and STS) and CS, a series of linear regression analyses were conducted. Based on the theoretical foundations of the MOPR training, we hypothesized that a broader OAZ would predict lower CF (both BO and STS) and higher CS.

#### 3.6.1. Compassion Satisfaction

A linear regression was conducted to examine the impact of the OAZ on CS. The model was significant, *F*(1, 71) = 16.23, *p* < 0.001, accounting for 18.6% of the variance in CS (*R^2^* = 0.186). The OAZ was a significant positive predictor (*β* = 3.101, *SE* = 0.770, *t*(71) = 4.03, *p* < 0.001) indicating that broader OAZ scores were associated with higher Compassion Satisfaction scores.

#### 3.6.2. Burnout

A simple linear regression was conducted to examine whether pre–post MOPR differences in the OAZ predict the same differences in BO. The regression model was significant, *F*_(1, 71)_ = 30.144, *p* < 0.001, with *R*^2^ = 0.298. This indicates that the OAZ explains approximately 29.8% of the variance in BO. The regression coefficient for the OAZ was significant, *β* = −4.246, *SE* = −0.773, *t*(71) = −5.490, *p* < 0.001. The negative sign of the coefficient indicates that higher values of OAZ scores are associated with lower values of BO.

#### 3.6.3. Secondary Traumatic Stress

A simple linear regression was conducted to investigate whether the OAZ predicts STS. The regression model was statistically significant, *F*_(1, 71)_ = 10.813, *p* = 0.002, with *R*^2^ = 0.132, indicating that the OAZ explains 13.2% of the variance in STS. The standardized regression coefficient for the OAZ was significant, *β =* −0.364, SE = 0.774, *t*(71) = −3.288, *p* = 0.002. This suggests that higher values of OAZ scores are associated with lower values of STS.

In summary, the results indicate that pre–post MOPR differences in the OAZ significantly predict changes in Compassion Satisfaction, Burnout, and Secondary Traumatic Stress, explaining 18.6%, 29.8%, and 13.2% of the variance, respectively. These findings highlight the importance of the OAZ as a predictor of job satisfaction and psychological distress outcomes.

### 3.7. Summary of Key Findings

The results of this study highlight the potential benefits of the MOPR program for healthcare professionals. Participants showed significant improvements in professional quality of life, including increased Compassion Satisfaction and reduced Burnout and Secondary Traumatic Stress. Mindfulness skills, particularly in Observing, Describing, Acting with Awareness, and Non-reactivity, also improved significantly. Furthermore, participants demonstrated enhanced arousal regulation, characterized by an expanded Optimal Arousal Zone and reduced hyperarousal and hypoarousal stress responses. These changes were accompanied by significant gains in psychological well-being, particularly in Self-Acceptance and Positive Relationships with others. Regression analyses further revealed that the breadth of the Optimal Arousal Zone was a strong predictor of improvements in professional quality of life outcomes. Collectively, these findings underscore the potential of the MOPR program to foster resilience and psychological well-being among healthcare providers, aligning with its integrative design of mindfulness-based and self-regulation interventions.

## 4. Discussion

The findings of this pilot study underscore the effectiveness of the Mindfulness-Oriented Professional Resilience (MOPR) program in enhancing professional quality of life, mindfulness, arousal modulation, and psychological well-being among healthcare workers. The statistically significant increases in Compassion Satisfaction (CS) and reductions in Burnout (BO) and Secondary Traumatic Stress (STS) indicate that the MOPR program effectively addresses both the positive and negative aspects of caregiving, as conceptualized within the Professional Quality of Life (ProQOL) model. This outcome aligns with existing research showing that structured interventions focused on resilience and self-care can significantly alleviate Compassion Fatigue (CF) among healthcare providers, particularly those frequently exposed to trauma and emotional distress [2,61].

The observed improvements across multiple facets of mindfulness—including Observing, Describing, Acting with Awareness, and Non-reacting—reflect the mindfulness-oriented design of the MOPR program, which emphasized non-reactive awareness and present-moment focus. These facets of mindfulness are crucial for healthcare professionals managing the emotional demands of caregiving, as they foster a more adaptive, less reactive response to stressors [62,63]. Enhancements in these mindfulness areas may directly contribute to reducing CF by enabling healthcare providers to observe their experiences with greater objectivity, articulate their emotions more effectively, and remain attentive without becoming overwhelmed [64]. Specifically, the ability to Act with Awareness could help staff in high-stress situations to remain focused and present, thereby reducing automatic, stress-fueled reactions. Similarly, improvements in non-reactivity may allow professionals to approach patient distress with greater emotional stability, preventing the accumulation of STS. Together, these mindfulness facets create a foundation of resilience, helping healthcare providers navigate the demands of their roles with a balanced, sustainable approach that may mitigate the onset of CF.

The MOPR program also demonstrated significant effects on arousal modulation, as evidenced by increases in the Optimal Arousal Zone (OAZ) and reductions in maladaptive stress responses, including hyperarousal (Fight/Flight, Freeze) and hypoarousal (Feigned Death). This outcome suggests that the mindfulness practices and vagal stimulation exercises included in the MOPR program, such as resonance frequency breathing, provided participants with effective tools for autonomic regulation [65,66]. Regression analysis findings further substantiated that arousal regulation, represented by the breadth of the OAZ, serves as a critical factor in enhancing professional quality of life. A broader OAZ was significantly associated with reduced levels of BO and STS, alongside increased levels of CS. These results are consistent with the hypothesis that the MOPR program’s integrative framework—combining bottom-up strategies (e.g., mindfulness and self-regulation exercises) with top-down approaches (e.g., guided reflection and psychoeducation)—facilitates meaningful improvements. Such improvements are particularly salient for healthcare professionals, who frequently encounter heightened arousal due to continuous exposure to patient suffering. This underscores the role of physiological regulation as a foundational component of resilience-focused interventions, emphasizing its importance in mitigating distress and fostering sustainable professional well-being.

The observed broadening of the OAZ has significant clinical implications for healthcare professionals. From a psychophysiological perspective, an expanded OAZ is associated with reduced reactivity to stress, both in hyperarousal (e.g., fight-or-flight responses) and hypoarousal (e.g., shutdown or Feigned Death states) [53]. This reduction in reactivity facilitates a greater sense of perceived safety, which is critical for enabling the activation of the social engagement system [54]. The social engagement system, involving the vagus nerve, plays a pivotal role in fostering interpersonal connection, emotional regulation, and resilience—key factors for effective caregiving and teamwork in high-stress environments.

Psychological well-being improvements, particularly in “Self-Acceptance” and “Positive Relations with others”, further indicate that the MOPR program facilitated a more balanced, resilient approach to personal and professional challenges. Although other dimensions, such as “Autonomy” and “Environmental Mastery”, did not reach statistical significance, the overall trend suggests a positive shift in participants’ well-being [67,68]. The focus on self-compassionate attitudes and on value-driven reflection through Acceptance and Commitment Therapy (ACT) may have contributed to these gains, encouraging participants to align their actions with their core values, fostering both personal and interpersonal growth and acceptance [69,70].

### Study Limitations

This study showed several limitations that should be addressed in future research. First, the sample was predominantly composed of female participants, which may restrict the generalizability of the findings to male healthcare workers. Future studies should strive for a more balanced gender representation to better evaluate potential differences in intervention outcomes across genders. Moreover, the voluntary nature of the recruitment process and the initial emphasis on psychologists in this study represent important limitations that warrant further discussion. While the recruitment approach facilitated program sustainability by identifying potential future trainers, it may have introduced selection bias, as participants were likely to have a pre-existing interest in or familiarity with the concepts of mindfulness and psychological well-being. This could limit the generalizability of the findings to the broader population of healthcare professionals, particularly those who may not prioritize or seek out such training opportunities. To address these limitations in future research, we recommend adopting recruitment strategies that ensure broader representation across healthcare professions and minimize the influence of self-selection. Randomized invitations, stratified sampling, or active outreach to under-represented groups may help achieve a more diverse participant pool.

Second, the absence of a control group prevents definitive conclusions about the causal impact of the intervention, as improvements could partially reflect natural changes over time. Future studies should prioritize randomized controlled trials with larger sample sizes to strengthen the internal validity and generalizability of the findings. Additionally, the reliance on post-intervention measurements without extended follow-up assessments limits the ability to evaluate the long-term sustainability of the observed benefits. While the immediate positive effects of the MOPR program are promising, future studies should incorporate follow-up assessments conducted at multiple time points after the intervention to determine whether these improvements persist over time.

Third, data on participants’ length of time in their profession were not collected, limiting the possibility to investigate how professional experience might influence the intervention’s effects. Future research should include this variable to explore its potential role as a moderator of intervention outcomes.

Finally, comparative studies are recommended to assess the effectiveness of MOPR against alternative interventions, such as other mindfulness-based or resilience-building programs, to identify relative advantages and specific mechanisms of action. Future research should also investigate the adaptability and impact of MOPR in diverse healthcare settings, including varying professional roles, organizational cultures, and resource levels. Such efforts would enhance our understanding of the program’s scalability and its potential to address the unique challenges faced by healthcare providers in different contexts.

## 5. Conclusions

In conclusion, the MOPR program offers a structured, evidence-based approach to mitigating Compassion Fatigue in healthcare professionals. By integrating mindfulness practices, arousal modulation techniques, and resilience-building exercises, the MOPR training demonstrates potential as a proactive, adaptable intervention that supports sustained well-being and resilience among healthcare workers in high-stress environments.

## Figures and Tables

**Table 1:** Core themes of the MOPR program with corresponding theoretical frameworks and intervention models.

Core Themes	Theoretical Frameworks and Intervention Models
Mindfulness meditation	- Mindfulness-Based Stress Reduction (MBSR) [48] - Mindfulness-Oriented Meditation (MOM) [46,49]
Mindfulness-based approaches	- Acceptance and Commitment Therapy (ACT) [50,51] - Self-Compassion (SC) [52]
Compassion satisfaction (CS) and Compassion fatigue (CF)	- Professional Quality of Life (ProQOL) Model [3,7] - Accelerated Recovery Program (ARP) [43,47]
Psychological trauma	- Sensorimotor Psychotherapy [53] - Polyvagal Theory [54]
Vagal stimulation and autoregulation	Heart Rate Variability (HRV) Biofeedback [55,56]

**Table 2:** Structure of the six MOPR program sessions.

Session	Title	Psychoeducation	Guided Work Reflection	Mindfulness Practices	Self-Regulation Exercises
1	Breath	Mindfulness; Resonance frequency breathing	Professional values	Mindful breathing meditation	Diaphragmatic breathing at resonance frequency
2	Body	Compassion fatigue; Arousal modulation model	Professional goals	Body scan meditation	Stretching (eyes and neck)
3	Mind	Psychological trauma; Polyvagal theory	Committed action at work	Mind awareness meditation	Stretching (sternocleidomastoid and trapezius)
4	Equanimity	From reaction to response	Work-related obstacles	Comprehensive meditation (breath, body scan, mind awareness)	Stretching (psoas)
5	Relationships	Compassion; Self-compassion	Experiential avoidance loops at work	Loving-kindness exercises	Soothing touch
6	Integration	Resilience; Medically unexplained symptoms; Lifestyle	Individual resilience plan		

**Table 3:** Demographic, educational, professional, and health-related characteristics of MOPR participants.

Variable	Category	N ^1^	Percentage (%)
Gender	Male	10	14%
	Female	63	86%
*Educational level*			
Degree	High school	10	14%
	Bachelor’s degree	15	21%
	Master’s degree	19	26%
	PhD or Postgraduate diploma	29	40%
*Professional background*			
Healthcare professionals	Medical doctor	6	8%
	(M = 2, F = 4; Age 52.33, SD = 10.23)		
	Psychologist	38	52%
	(M = 4, F = 34; Age 49.24, SD = 7.78)		
	Nurse	18	25%
	(M = 3, F = 15; Age 49.61, SD = 10.79)		
	Healthcare assistant	2	3%
	(M = 0, F = 2; Age 45.50, SD = 14.85)		
	Rehabilitation therapist	8	11%
	(M = 1, F = 7; Age 40.50, SD = 10.28)		
	Biomedical laboratory technician	1	1%
	(M = 1, F = 0; Age 54)		
*Health and wellness characteristics*			
Physical Activity Level	Physically Active Person	22	30%
	Partially Active Person	37	51%
	Sedentary Person	14	19%
Meditation	Yes	16	22%
	No	57	78%
Breathing Exercises	Yes	18	25%
	No	55	75%
Mental Health Diagnosis	Yes	4	5%
	No	69	95%
Psychotropic Medication Use	Yes	4	5%
	No	69	95%

N ^1^ = Number of participants; M = Male, F = Female.

**Table 4:** Changes in outcome measures across time.

Scale	Time Point	*M*	*SD*	ANOVA *F*	*p*-Value ^1^	*η_p_* ^2^
*Professional Quality of Life (ProQOL)*						
Compassion satisfaction	Pre	37.47	5.85	16.84	<0.001	0.19
	Post	39.18	5.84			
Burnout	Pre	19.43	4.83	9.21	0.003	0.11
	Post	18.06	4.92			
Secondary traumatic stress	Pre	21.01	5.12	22.57	<0.001	0.24
	Post	19.08	5.43			
*Five Facet Mindfulness Questionnaire—Short Form (FFMQ-SF)*						
Observing	Pre	14.08	3.59	9.13	0.003	0.11
	Post	15.36	3.44			
Describing	Pre	17.78	3.90	7.04	0.01	0.09
	Post	18.90	3.85			
Acting with awareness	Pre	17.21	4.18	13.83	<0.001	0.16
	Post	19.11	3.86			
Non-judging	Pre	16.69	4.64	0.57	0.454	0.01
	Post	17.10	4.82			
Non-reacting	Pre	15.01	3.44	10.77	0.002	0.13
	Post	16.14	3.20			
*Arousal Modulation Model Questionnaire (AMMQ)*						
Optimal arousal zone	Pre	2.74	0.69	22.28	<0.001	0.24
	Post	3.01	0.65			
Fight/flight	Pre	1.16	0.90	11.20	0.001	0.14
	Post	0.85	0.77			
Freeze	Pre	0.75	0.66	4.71	0.033	0.06
	Post	0.60	0.62			
Feigned death	Pre	1.00	0.73	5.98	0.017	0.08
	Post	0.82	0.72			
*Psychological Well-Being Scale—Short Form (PWB-SF)*						
Self-acceptance	Pre	13.85	3.17	6.35	0.014	0.08
	Post	14.62	2.90			
Autonomy	Pre	12.16	3.38	0.46	0.500	0.01
	Post	12.47	3.39			
Personal growth	Pre	16.21	2.20	0.99	0.324	0.01
	Post	16.44	2.15			
Positive relations with others	Pre	13.40	2.91	4.34	0.041	0.06
	Post	14.12	2.87			
Purpose in life	Pre	14.32	3.34	1.55	0.218	0.02
	Post	14.73	3.11			

^1^ Bonferroni-corrected *p*-value.

## Data Availability

The data supporting the findings of this study are not publicly available due to ethical and privacy restrictions but can be obtained from the corresponding author upon reasonable request.

## References

[1] Joinson C. (1992). Coping with compassion fatigue. Nursing.

[2] Patole S., Pawale D., Rath C. (2024). Interventions for Compassion Fatigue in Healthcare Providers—A Systematic Review of Randomised Controlled Trials. Healthcare.

[3] Figley C.R., Stamm B.H. (1999). Compassion fatigue: Toward a new understanding of the costs of caring. Secondary Traumatic Stress: Self-Care Issues for Clinicians, Researchers, and Educators.

[4] Figley C.R., Figley C.R. (1995). Compassion fatigue as secondary traumatic stress disorder: An overview. Compassion Fatigue: Coping with Secondary Traumatic Stress Disorder in Those Who Treat the Traumatized.

[5] Figley C.R., Kleber R.J., Kleber R.J., Figley C.R., Gersons B.P.R. (1995). Beyond the “victim”: Secondary traumatic stress. Beyond Trauma: Cultural and Societal Dynamics.

[6] Stamm B.H. (2009). Professional Quality of Life: Compassion Satisfaction and Fatigue Version 5 (ProQOL). https://proqol.org/proqol-measure.

[7] Stamm B.H. (2010). The Concise ProQOL Manual. https://proqol.org/proqol-manual.

[8] De Luca R., Bonanno M., Maggio M.G., Todaro A., Rifici C., Mento C., Muscatello M.R.A., Castorina M.V., Tonin P., Quartarone A. (2024). Compassion Fatigue in a Cohort of South Italian Nurses and Hospital-Based Clinical Social Workers Following COVID-19: A Cross-Sectional Survey. J. Clin. Med..

[9] Rayani A., Hannan J., Alreshidi S., Aboshaiqah A., Alodhailah A., Hakamy E. (2024). Compassion Satisfaction, Burnout, and Secondary Traumatic Stress among Saudi Nurses at Medical City: A Cross-Sectional Study. Healthcare.

[10] Lobo R., Kumar S.P., Tm R. (2024). Professional Quality of Life Among Mental Health Nurses: A Systematic Review and Meta-Analysis. Int. J. Ment. Health Nurs..

[11] Algamdi M. (2022). Prevalence of Oncology Nurses’ Compassion Satisfaction and Compassion Fatigue: Systematic Review and Meta-Analysis. Nurs. Open.

[12] Alharbi J., Jackson D., Usher K. (2019). Compassion Fatigue in Critical Care Nurses: An Integrative Review of the Literature. Saudi Med. J..

[13] Alipio J., Florendo M.G., Montilla M.G., Narvaez R.A. (2023). Compassion Fatigue in Oncology Nurses: An Integrative Review. World J. Cancer Oncol. Res..

[14] Hooper C., Craig J., Janvrin D.R., Wetsel M.A., Reimels E. (2010). Compassion Satisfaction, Burnout, and Compassion Fatigue Among Emergency Nurses Compared With Nurses in Other Selected Inpatient Specialties. J. Emerg. Nurs..

[15] Ortega-Campos E., Vargas-Román K., Velando-Soriano A., Suleiman-Martos N., Cañadas-de la Fuente G.A., Albendín-García L., Gómez-Urquiza J.L. (2020). Compassion Fatigue, Compassion Satisfaction, and Burnout in Oncology Nurses: A Systematic Review and Meta-Analysis. Sustainability.

[16] Garnett A., Hui L., Oleynikov C., Boamah S. (2023). Compassion Fatigue in Healthcare Providers: A Scoping Review. BMC Health Serv. Res..

[17] Gribben J.L., Kase S.M., Waldman E.D., Weintraub A.S. (2019). A Cross-Sectional Analysis of Compassion Fatigue, Burnout, and Compassion Satisfaction in Pediatric Critical Care Physicians in the United States. Pediatr. Crit. Care Med..

[18] Ruiz-Fernández M.D., Pérez-García E., Ortega-Galán Á.M. (2020). Quality of Life in Nursing Professionals: Burnout, Fatigue, and Compassion Satisfaction. Int. J. Environ. Res. Public Health.

[19] Adams R.E., Boscarino J.A., Figley C.R. (2006). Compassion Fatigue and Psychological Distress Among Social Workers: A Validation Study. Am. J. Orthopsychiatry.

[20] Casado T., Rosselló M.V., Cañas-Lerma A. (2023). Changes in Social Interventions after COVID-19: The Experience of Front-Line Social Workers. Soc. Sci..

[21] Craig C.D., Sprang G. (2010). Compassion Satisfaction, Compassion Fatigue, and Burnout in a National Sample of Trauma Treatment Therapists. Anxiety Stress Coping.

[22] Dehlin M., Lundh L.G. (2018). Compassion Fatigue and Compassion Satisfaction Among Psychologists: Can Supervision and a Reflective Stance Be of Help?. J. Pers.-Oriented Res..

[23] Watson V.C., Begun S. (2025). Burnout in Social Work: A Review of the Literature within the Context of COVID-19. Soc. Work Public Health.

[24] Campos i Arnal A., Galiana L., Sánchez-Ruiz J., Sansó N. (2024). Cross-Sectional Study of the Professional Quality of Life of Palliative Care Professionals during the COVID-19 Pandemic. Healthcare.

[25] Han S.-J., Lee S.-Y., Kim S.-E. (2023). An Exploratory Study of Psychological Distress, Professional Quality of Life, Effort-Reward Imbalance, and Turnover Intention of Hospital Nurses during the COVID-19 Pandemic. Healthcare.

[26] Rania N., Coppola I., Brucci M. (2023). Mental Health and Quality of Professional Life of Healthcare Workers: One Year after the Outbreak of the COVID-19 Pandemic. Sustainability.

[27] Romito B.T., Okoro E.N., Ringqvist J.R.B., Goff K.L. (2020). Burnout and Wellness: The Anesthesiologist’s Perspective. Am. J. Lifestyle Med..

[28] Vittori A., Marinangeli F., Bignami E.G., Simonini A., Vergallo A., Fiore G., Petrucci E., Cascella M., Pedone R. (2022). Analysis on Burnout, Job Conditions, Alexithymia, and Other Psychological Symptoms in a Sample of Italian Anesthesiologists and Intensivists, Assessed Just before the COVID-19 Pandemic: An AAROI-EMAC Study. Healthcare.

[29] West C.P., Dyrbye L.N., Shanafelt T.D. (2018). Physician Burnout: Contributors, Consequences, and Solutions. J. Intern. Med..

[30] Lee T., Becerra B.J., Becerra M.B. (2023). “*Seems Like There Is No Stopping Point at All Whatsoever*”: A Mixed-Methods Analysis of Public Health Workforce Perception on COVID-19 Pandemic Management and Future Needs. Int. J. Environ. Res. Public Health.

[31] Armstrong J.W., Turne L.N. (2022). Mindfulness-based interventions to reduce stress and burnout in nurses: An integrative review. Br. J. Ment. Health Nurs..

[32] Dominguez-Rodriguez A., Martínez-Arriaga R.J., Herdoiza-Arroyo P.E., Bautista-Valerio E., de la Rosa-Gómez A., Castellanos Vargas R.O., Lacomba-Trejo L., Mateu-Mollá J., Lupercio Ramírez M.d.J., Figueroa González J.A. (2022). E-Health Psychological Intervention for COVID-19 Healthcare Workers: Protocol for its Implementation and Evaluation. Int. J. Environ. Res. Public Health.

[33] Katzman J.W., Tomedi L.E., Pandey N., Richardson K., Xenakis S.N., Heines S., Grabbe L., Magdaleno Y., Mehta A., Welton R. (2024). Caring for the Caregivers: Improving Mental Health among Health Professionals Using the Behavioral Health Professional Workforce Resilience ECHO Program. Healthcare.

[34] Benavides-Gil G., Martínez-Zaragoza F., Fernández-Castro J., García-Campayo J., Demarzo M., Miralles C., Pascual A., Oliván-Blázquez B. (2024). Mindfulness-Based Interventions for Improving Mental Health of Frontline Healthcare Professionals during the COVID-19 Pandemic: A Systematic Review. Syst. Rev..

[35] Lomas T., Medina J.C., Ivtzan I., Fu C.H., Hart R., Eiroa-Orosa F.J. (2019). A Systematic Review and Meta-Analysis of the Impact of Mindfulness-Based Interventions on the Well-Being of Healthcare Professionals. Mindfulness.

[36] Ong N.Y., Teo F.J.J., Ee J.Z.Y., Yau C.E., Thumboo J., Tan H.K., Ng Q.X. (2024). Effectiveness of Mindfulness-Based Interventions on the Well-Being of Healthcare Workers: A Systematic Review and Meta-Analysis. Gen. Psychiatry.

[37] Lalani K., O’Neal M., Joannou S.L., Gopal B., Champagne-Langabeer T. (2023). Helping Frontline Workers in Texas—A Framework for Resource Development. Int. J. Environ. Res. Public Health.

[38] Pérez V., Menéndez-Crispín E.J., Sarabia-Cobo C., de Lorena P., Fernández-Rodríguez A., González-Vaca J. (2022). Mindfulness-Based Intervention for the Reduction of Compassion Fatigue and Burnout in Nurse Caregivers of Institutionalized Older Persons with Dementia: A Randomized Controlled Trial. Int. J. Environ. Res. Public Health.

[39] Sulosaari V., Unal E., Cinar F.I. (2022). The effectiveness of mindfulness-based interventions on the psychological well-being of nurses: A systematic review. Appl. Nurs. Res..

[40] Lipsa J.M., Rajkumar E., Gopi A., Romate J. (2024). Effectiveness of Psychological Interventions for Compassion Fatigue: A Systematic Review and Meta-Analysis. J. Occup. Health.

[41] Lluch C., Galiana L., Doménech P., Sansó N. (2022). The Impact of the COVID-19 Pandemic on Burnout, Compassion Fatigue, and Compassion Satisfaction in Healthcare Personnel: A Systematic Review of the Literature Published during the First Year of the Pandemic. Healthcare.

[42] Gentry J.E. (2002). Compassion Fatigue: A Crucible of Transformation. J. Trauma Pract..

[43] Gentry J.E., Baggerly J., Baranowsky A. (2004). Training-as-Treatment: Effectiveness of the Certified Compassion Fatigue Specialist Training. Int. J. Emerg. Ment. Health.

[44] Orrù G., Marzetti F., Conversano C., Vagheggini G., Miccoli M., Ciacchini R., Panait E., Gemignani A. (2021). Secondary Traumatic Stress and Burnout in Healthcare Workers during COVID-19 Outbreak. Int. J. Environ. Res. Public Health.

[45] Kriakous S.A., Elliott K.A., Lamers C., Owen R. (2021). The Effectiveness of Mindfulness-Based Stress Reduction on the Psychological Functioning of Healthcare Professionals: A Systematic Review. Mindfulness.

[46] Campanella F., Crescentini C., Urgesi C., Fabbro F. (2014). Mindfulness-Oriented Meditation Improves Self-Related Character Scales in Healthy Individuals. Compr. Psychiatry.

[47] Gentry J.E., Baranowsky A.B., Dunning K., Figley C.R. (2002). The Accelerated Recovery Program (ARP) for Compassion Fatigue. Treating Compassion Fatigue.

[48] Kabat-Zinn J. (1990). Full Catastrophe Living: Using the Wisdom of Your Body and Mind to Face Stress, Pain, and Illness.

[49] Fabbro F., Crescentini C. (2016). La meditazione orientata alla mindfulness (MOM) nella ricerca psicologica. Ricerche di Psicologia.

[50] Hayes S.C., Lillis J. (2012). Acceptance and Commitment Therapy.

[51] Polk K.L., Schoendorff B. (2014). The ACT Matrix: A New Approach to Building Psychological Flexibility across Settings and Populations.

[52] Neff K.D., Germer C.K., Siegel R.D. (2012). The Science of Self-Compassion. Wisdom and Compassion in Psychotherapy: Deepening Mindfulness in Clinical Practice.

[53] Ogden P., Minton K., Pain C. (2006). Trauma and the Body: A Sensorimotor Approach to Psychotherapy.

[54] Porges S.W. (2011). The Polyvagal Theory: Neurophysiological Foundations of Emotions, Attachment, Communication, and Self-Regulation.

[55] Khazan I. (2019). Biofeedback and Mindfulness in Everyday Life: Practical Solutions for Improving Your Health and Performance.

[56] Lehrer P.M., Gevirtz R. (2014). Heart rate variability biofeedback: How and why does it work?. Front. Psychol..

[57] Palestini L., Prati G., Pietrantoni L., Cicognani E. (2009). La Qualità della Vita Professionale nel Lavoro di Soccorso: Un Contributo alla Validazione Italiana della Professional Quality of Life Scale (ProQOL). Psicoter. Cogn. Comport..

[58] Iani L., Lauriola M., Cafaro V. (2020). The Assessment of Mindfulness Skills: The “What” and the “How”. J. Ment. Health.

[59] Ruini C., Ottolini F., Rafanelli C., Ryff C.D., Fava G.A. (2003). Italian Validation of Psychological Well-Being Scales (PWB). Riv. Psichiatr..

[60] Sirigatti S., Stefanile C., Giannetti E., Iani L., Penzo I., Mazzeschi A. (2009). Assessment of Factor Structure of Ryff’s Psychological Well-Being Scales in Italian Adolescents. Giunti Organ. Spec..

[61] Cocker F., Joss N. (2016). Compassion Fatigue among Healthcare, Emergency and Community Service Workers: A Systematic Review. Int. J. Environ. Res. Public Health.

[62] Lomas T., Medina J.C., Ivtzan I., Rupprecht S., Eiroa-Orosa F.J. (2018). A Systematic Review of the Impact of Mindfulness on the Well-Being of Healthcare Professionals. J. Clin. Psychol..

[63] Tripathi S.K., Mulkey D.C. (2023). Implementing Brief Mindfulness-Based Interventions to Reduce Compassion Fatigue. Crit. Care Nurse.

[64] Burton A., Burgess C., Dean S., Koutsopoulou G.Z., Hugh-Jones S. (2017). How Effective are Mindfulness-Based Interventions for Reducing Stress Among Healthcare Professionals? A Systematic Review and Meta-Analysis. Stress Health.

[65] Pascoe M.C., Thompson D.R., Jenkins Z.M., Ski C.F. (2017). Mindfulness Mediates the Physiological Markers of Stress: Systematic Review and Meta-Analysis. J. Psychiatr. Res..

[66] Steffen P.R., Austin T., DeBarros A., Brown T. (2017). The Impact of Resonance Frequency Breathing on Measures of Heart Rate Variability, Blood Pressure, and Mood. Front. Public Health.

[67] Wilkie L., Fisher Z., Kemp A.H. (2022). The Complex Construct of Wellbeing and the Role of Vagal Function. Front. Integr. Neurosci..

[68] Nykliček I., Vingerhoets A., Zeelenberg M. (2011). Emotion Regulation and Well-Being.

[69] Paiva-Salisbury M.L., Schwanz K.A. (2022). Building Compassion Fatigue Resilience: Awareness, Prevention, and Intervention for Pre-Professionals and Current Practitioners. J. Health Serv. Psychol..

[70] Rushforth A., Durk M., Rothwell-Blake G.A.A., Kirkman A., Ng F., Kotera Y. (2023). Self-Compassion Interventions to Target Secondary Traumatic Stress in Healthcare Workers: A Systematic Review. Int. J. Environ. Res. Public Health.

